# Supplementary material for: The model diatom Phaeodactylum tricornutum provides insights into the diversity and function of microeukaryotic DNA methyltransferases
Source: Commun Biol. 2023 Mar 9;6:253. doi: 10.1038/s42003-023-04629-0 (PMC9998398; doi:10.1038/s42003-023-04629-0)
Supplement: Supplementary file 2 — Supplementary Information [file 42003_2023_4629_MOESM2_ESM.pdf]

Supplementary Figure 1: Summary cladogram of phylogenetic relationships between DNMTs

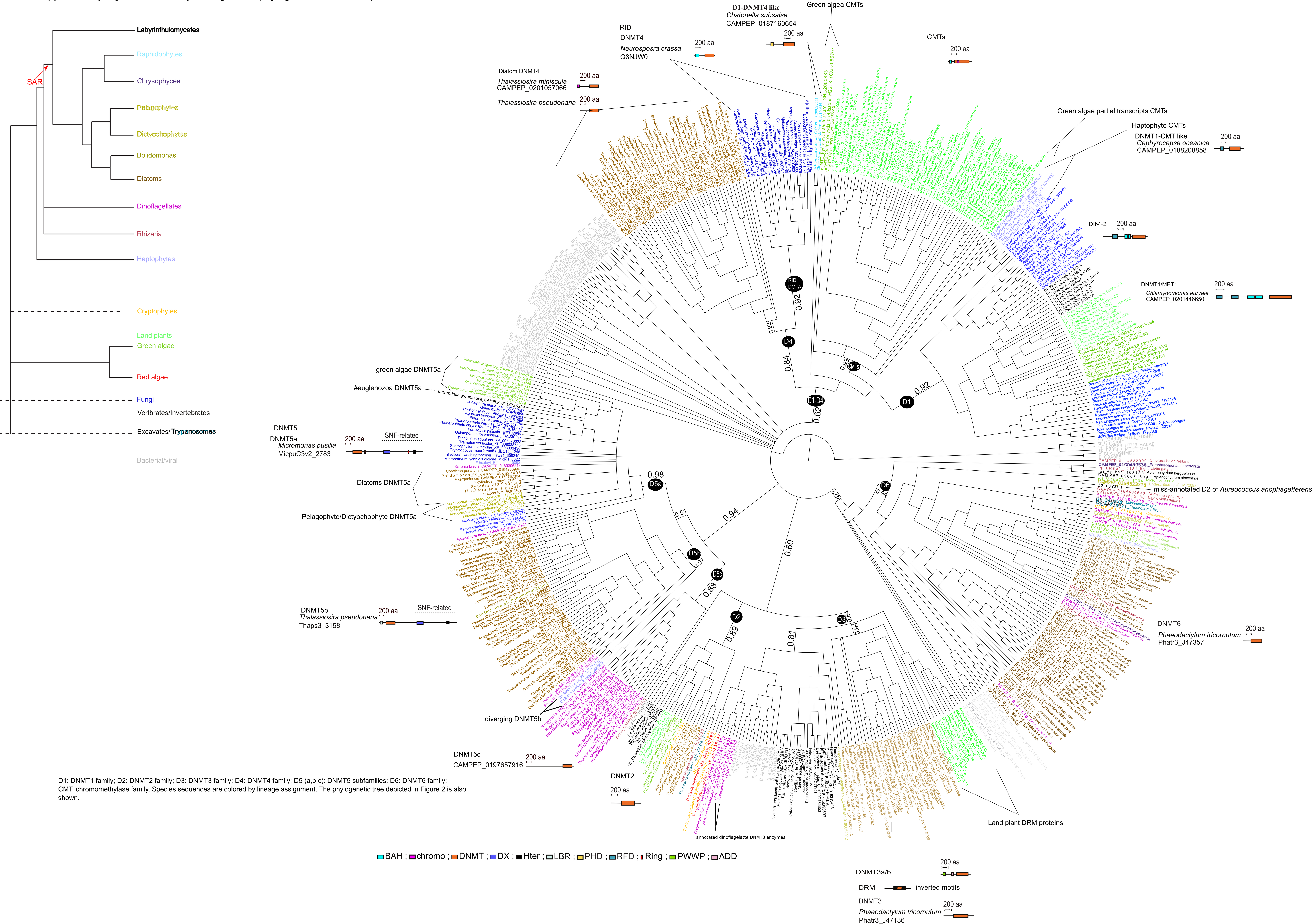

D1: DNMT1 family; D2: DNMT2 family; D3: DNMT3 family; D4: DNMT4 family; D5 (a,b,c): DNMT5 subfamilies; D6: DNMT6 family; CMT: chromomethylase family. Species sequences are colored by lineage assignment. The phylogenetic tree depicted in Figure 2 is also shown.

Suppplementary Figure 2: Alignment of the DNMT domain of representative DNMT5 proteins

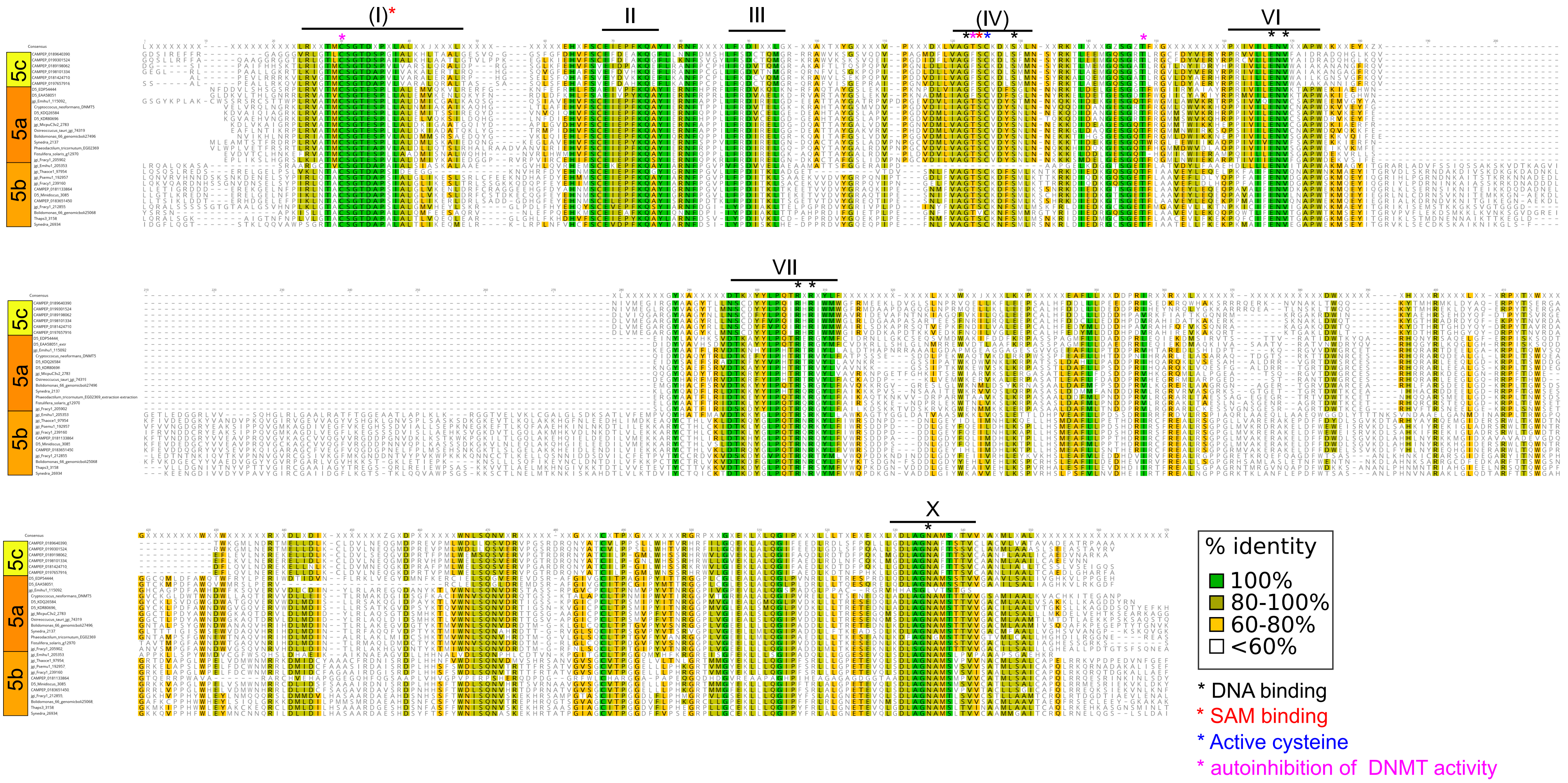

DNMT motifs are labelled using roman numerals. Motifs put in brackets are divergent compared to other DNMTs. An annotation is proposed for the motif I: TxCSGTD(A/S)P and IV: TSC; that are highly divergent compared to other DNMT motifs I (DXFXGXG) and IV (PCQ); based on their conservation in other DNMT5s and their position relatively to the other conserved DNMT motifs. Other motifs are well conserved and amino acids with DNA binding function and SAM binding activity are annotated accordingly.

Supplementary Figure 3: Sequencing of the DNMT5 CRISPR/Cas9 edited region in the DNMT5 KO cell lines and WT

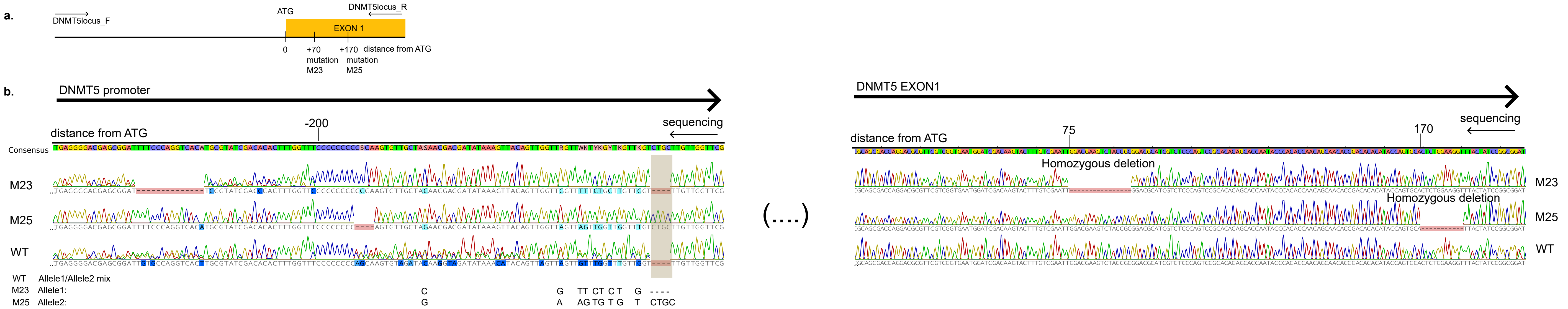

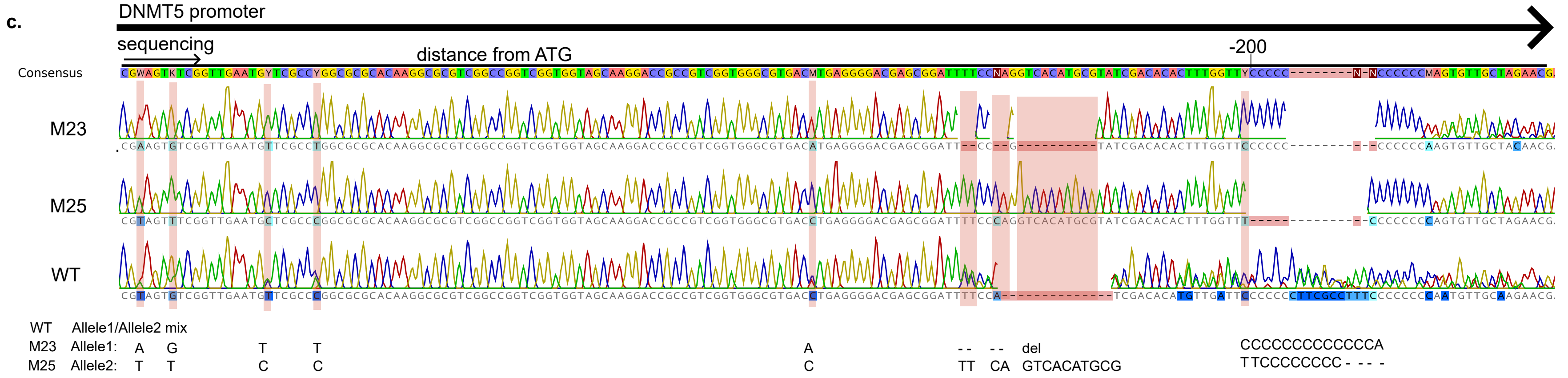

a. Schematic view of the DNMT5 locus showing mutation sites in M23 and M25 cell lines. **b.** Right panel: Schematic representation of the sequencing using the primer DNMT5locus_R. Homozygous mutations are generated by CRISPR/Cas9 in M23 and M25 cell lines at two independent target sequences. In M25, the mutation consists in 16 bp out of frame deletion around the CRISPR/Cas9 cutting sites that generates a loss of amino acids from position 28 to 34 leading to a premature STOP codon at amino acid 280. The M23 cell line has a 11 bp out of frame deletion that generates a loss of amino acids 58 to 60/61 followed by a premature STOP codon at amino acid position 179 -180. Left panel: sequencing tracks extending to the DNMT5 promoter region. The WT sequence is a mixture of two alleles found in M23 and M25 that are homozygous for all polymorphisms detected. **c.** as for **b.** using the DNMT5locus_F primer. The WT is a mixture of two alleles found in the DNMT5 mutants that are also homozygous.

Supplementary Figure 4 : DNMT5:KO cell lines

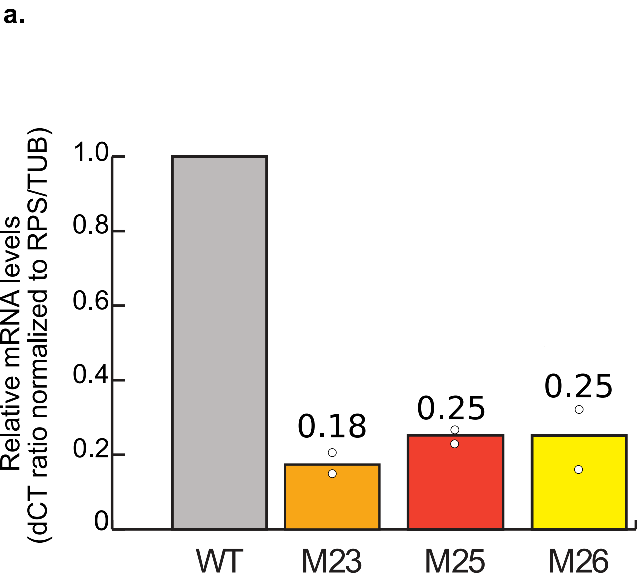

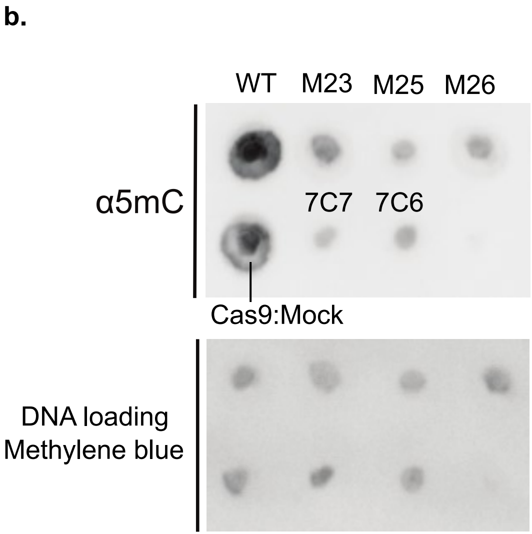

a. Quantitative PCR analysis of DNMT5 mRNA levels in the mutants compared to the reference Pt1 8.6 line (WT). Average fold loss is calculated by the ratio of CTs, normalized on the RPS and TUB genes (see Methods section), between mutants and WT. Normalized ratios were then averaged on biological replicates (n=2) per line (*2 technical replicates per biological replicate) for 5 primers targeting all the DNMT5 transcripts. DNMT5:KO M26 is an independent DNMT5:KO mutant showing a deletion at the same position of DNMT5:KO M23 and is not further described in this manuscript **b.** Dot blot analysis of DNMT5 mutants compared to the Pt1 8.6 reference line (WT) and the Cas9:Mock control. 7C4 and 7C6 are DNMT5:KOs mutants that were not further used in this study. No DNA methylation, compared to the reference strain, in any DNMT5:KO mutant could be detected.

Supplementary Figure 5 : Uncropped version of the dot blot shown in the Supplementary Figure 4b

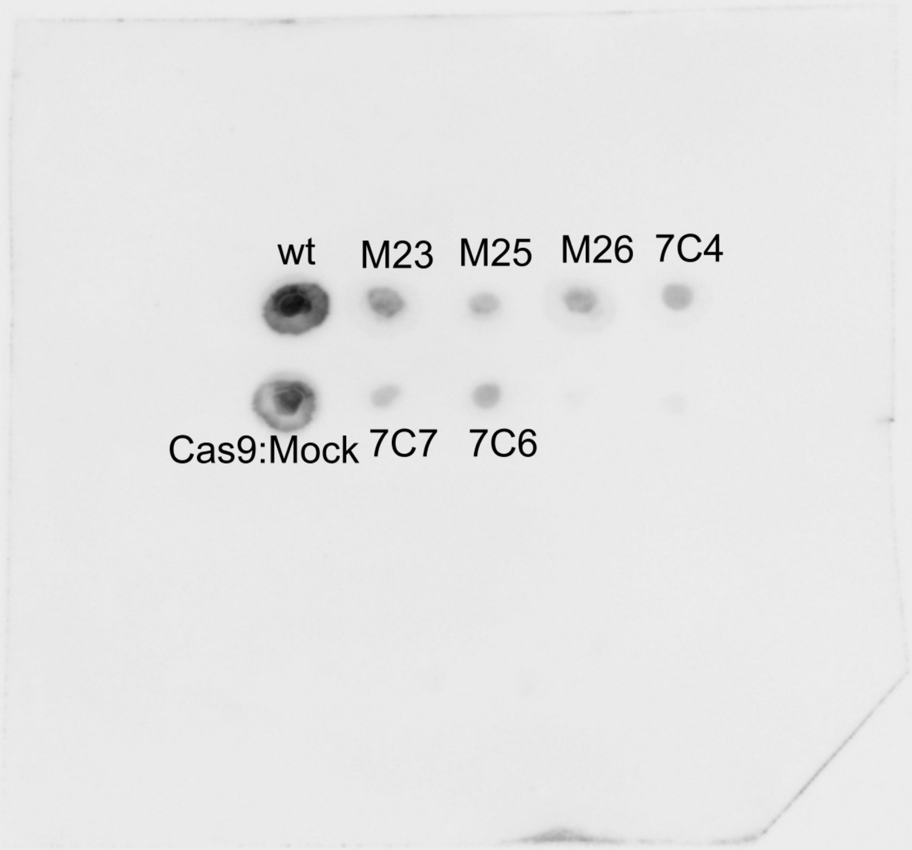

Dot blot depicting the DNMT5 mutants, the reference cell line Pt1 8.6 (WT) and the Cas9:Mock control. 7C4, 7C6 and 7C7 are DNMT5:KOs that were not used in this study. No DNA methylation, compared to the reference strain, in any DNMT5:KO mutant could be detected.

Supplementary Figure 6: Bisulfite sequencing features in the reference Pt1 8.6 and DNMT5:KO cell lines (M23, M25)

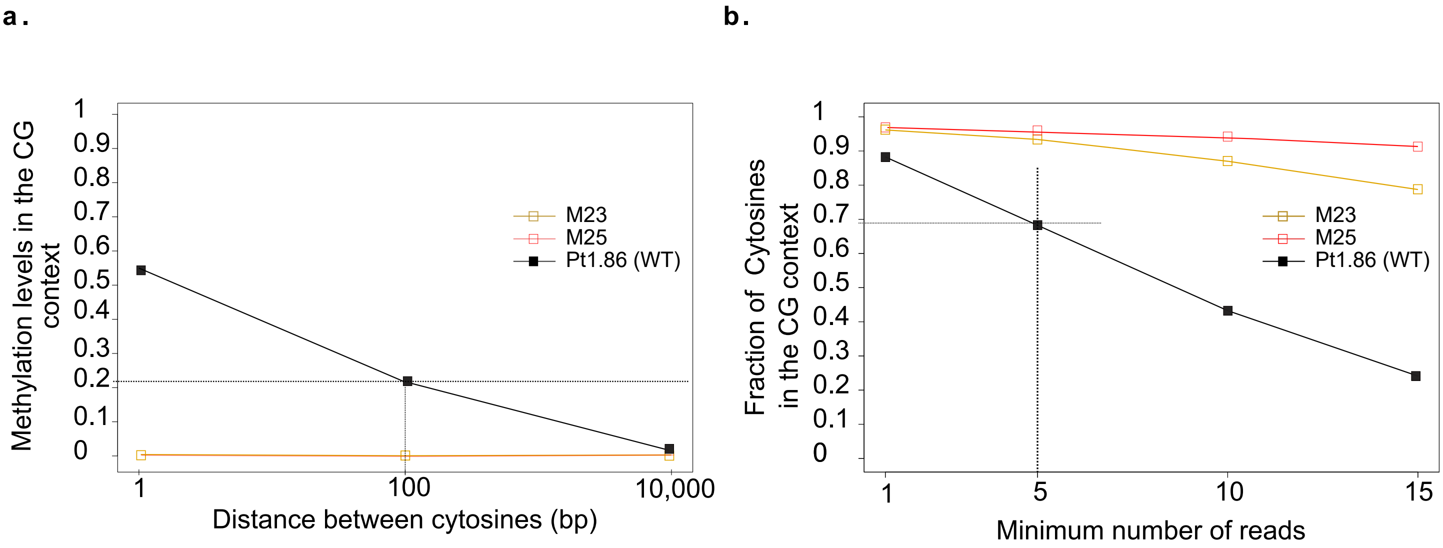

a. CG DNA methylation levels related to distance between cytosines in the reference Pt1 8.6 and DNMT5:KOs (M23, M25). DNA methylation levels sharply decline after 100 bp distance in the reference strain suggesting a sparse methylation pattern. No DNA methylation is found in DNMT5:KOs. **b.** Cytosine Coverage, after bisulfite treatment and Illumina sequencing in Pt1 8.6 and DNMT5:KOs, show a deeper cytosine coverage for mutants. The number of covered cytosines quickly drop in the reference

Supplementary Figure 7 : Quantitative PCR analysis of selected up and downregulated genes

a.

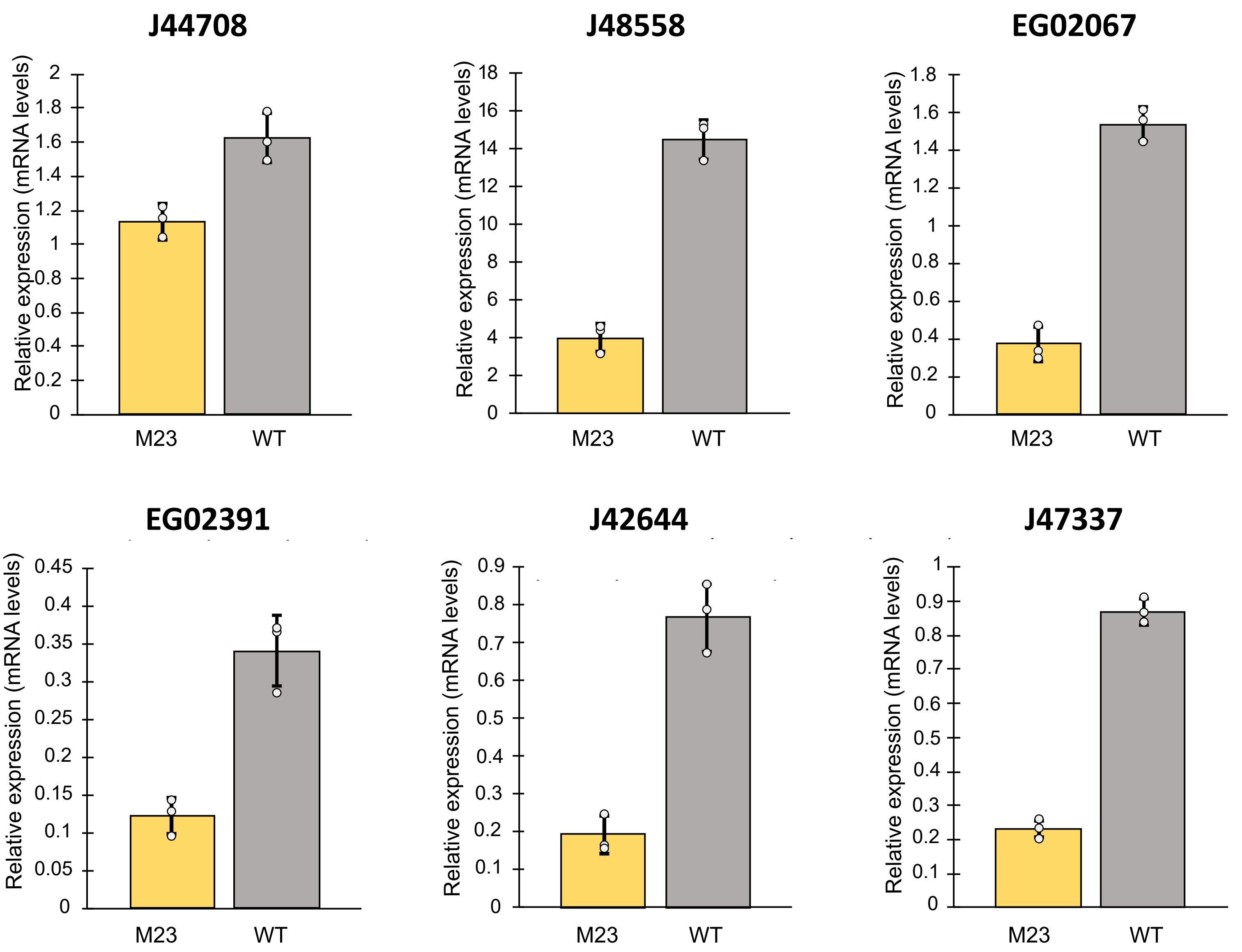

b.

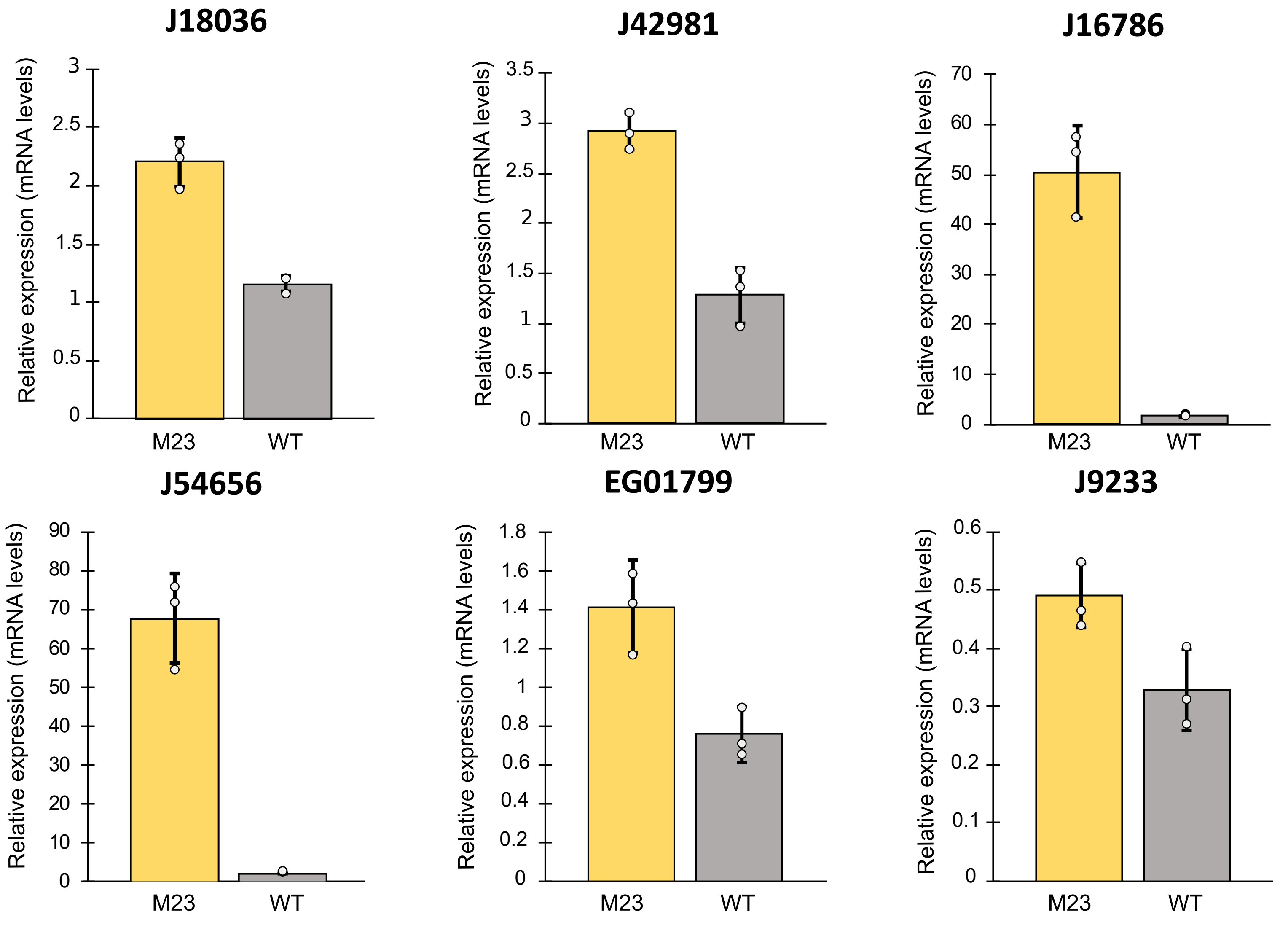

a. Quantitative PCR analysis of mRNA levels of downregulated genes in the DNMT5:KO M23 compared to the reference Pt1 8.6 line (WT). Average fold loss is calculated by the ratio of CTs, normalized on the RPS and TUB genes (see Methods section), between mutants and WT on technical replicates (n=3). Error bars represent the standard deviation between technical replicates. **b.** as for a. for upregulated genes. Biological functions of tested genes can be found in Supplementary Data 13 and 14.
