# Supplementary material for: The model diatom Phaeodactylum tricornutum provides insights into the diversity and function of microeukaryotic DNA methyltransferases
Source: Commun Biol. 2023 Mar 9;6:253. doi: 10.1038/s42003-023-04629-0 (PMC9998398; doi:10.1038/s42003-023-04629-0)
Supplement: Supplementary file 3 — Description of Additional Supplementary Files [file 42003_2023_4629_MOESM3_ESM.pdf]

Description of Additional Supplementary Files

Supplementary Data 1

Contains data relevant to Figure 1a. List of putative DNMTs fetched by HMMER in the MMETSP database and reciprocal best hits search in the phylogenetically optimized database described in Dorrell et al 61. When already reported in other studies, references were added. Enzymes found in the reference database and literature were added to the tree for comparison. References: De Mendoza A, Bonnet A, Vargas-Landin DB, Ji N, Hong F, Yang F, et al. Recurrent acquisition of cytosine methyltransferases into eukaryotic retrotransposons. Nat Commun 2018 ; Bewick, A. J. et al. The evolution of CHROMOMETHYLASES and gene body DNA methylation in plants. Genome Biol. 18, (2017).

Supplementary Data 2

Contains data relevant to Figure 1a. Summary of DNMTs found in microeukaryotes at the species level. Presence “1”, absence “x” of at least one DNMT transcript or gene per species.

Supplementary Data 3

Contains data relevant to Figure 2. Summary of DNMTs found per diatom species. The figure indicates the number of putative paralogues for each gene family in each species. The common ancestor of diatoms likely possessed a DNMT3, DNMT6, DNMT5b and DNMT2 enzyme while DNMT5a is restricted to pennate diatoms. Iterative lineage specific loss of DNMTs is observed as between *Fistulifera solaris*, *F. cylindrus* and *P. tricornutum*. *P. tricornutum* (in red) is the reference species for epigenomics in diatoms which lacks DNMT5b and DNMT4 orthologues. c-chromodomain containing-DNMT4 ; g – enzyme found in annotated genomes ; L - DNMT5b proteins with laminin-B receptor domain.

Supplementary Data 4

Contains data relevant to Figure 1b. Representative microeukaryote enzymes used for the phylogenetic analysis (Figure 1a) and their structure as given by the eDAF pipeline.

Supplementary Data 5

Contains data relevant to Supplementary Figure 4a. qCPR analysis of DNMT5a in DNMT5:KO cell lines and Pt1 8.6 in two biological replicates (B1 and B2) for 5 pairs of primers spanning the SNF and DX domains (DNMT5_SNF, DNMT5_DX as well as the DNMT domain of DNMT5 (DNMT5_DNMT (1); DNMT5_DNMT (2);DNMT5_DNMT (3)).

Supplementary Data 6

Contains data relevant to Figure 3a and b. Methylation levels of genes and TEs in the reference strain and overlap with common hypoDMRs in DNMT5:KO cell lines (M23, M25). For each gene and TE, the number of methylated cytosines and associated average DNA methylation levels are reported. Methylated and unmethylated read counts are also shown.

Supplementary Data 7

Contains data relevant to Figure 3c. Summary of DMR analysis in the DNMT5:KO M23 cell line. DMR coordinates, number of methylated reads/unmethylated reads and pooled percentages of DNA methylation levels are shown. loss = hypoDMRs, gain = hyperDMRs.

Supplementary Data 8

Contains data relevant to Figure 3c. Summary of DMR analysis in the DNMT5:KO M25 cell line. DMR coordinates, number of methylated reads/unmethylated reads and pooled percentages of DNA methylation levels are shown. loss = hypoDMRs, gain = hyperDMRs.

Supplementary Data 9

Contains data relevant to Figure 3d. List of TEs and genes that overlap with common HypoDMRs.

Supplementary Data 10

Contains data relevant to Figure 3e. List of common HypoDMRs that overlap ("1") or don't overlap ("0") with different histone marks.

Supplementary Data 11

Contains data relevant to Figure 4. Summary of RNAseq analysis.

Supplementary Data 12

Contains data relevant to Figure 4d and 4e. TopGO results of biological process enrichment of upregulated TE-genes and genes in both DNMT5:KO cell lines.

Supplementary Data 13

Contains data relevant to Figure 4d and 4e. Summary and list of upregulated genes in the DNMT5:KO cell lines.

Supplementary Data 14

Contains data relevant to Figure 4f and 4g. Summary and list of downregulated genes in the DNMT5:KO cell lines.

Supplementary Data 15

Contains data relevant to Supplementary Figure7a and 7b. qCPR analysis of upregulated and downregulated genes in the DNMT5:KO M23 cell line.

Supplementary Data 16

Contains data relevant to Figure 5a. Relationship between DNA methylation levels and expression of TE-genes.

Supplementary Data 17

Contains data relevant to Figure 5e. The table shows the length of TEs overlapping with upregulated TE-genes that also intersect (or not) with common hypoDMRs.

Supplementary Data 18

Contains data relevant to Figure 5d. Long-LTR TEs that overlap with common hypoDMRs and that are upregulated in DNMT5:KO cell lines

Supplementary Data 19

List of primers used in the study.
