# Supplementary material for: The model diatom Phaeodactylum tricornutum provides insights into the diversity and function of microeukaryotic DNA methyltransferases
Source: Commun Biol. 2023 Mar 9;6:253. doi: 10.1038/s42003-023-04629-0 (PMC9998398; doi:10.1038/s42003-023-04629-0)
Supplement: Supplementary file 23 — Reporting Summary [file 42003_2023_4629_MOESM23_ESM.pdf]

nature portfolio

Corresponding author(s): Leila Tirichine, Fabio Rocha Jimenez Vieira, Ouardia Ait-Mohamed

Last updated by author(s): Feb 4, 2023

Reporting Summary

Nature Portfolio wishes to improve the reproducibility of the work that we publish. This form provides structure for consistency and transparency in reporting. For further information on Nature Portfolio policies, see our Editorial Policies and the Editorial Policy Checklist.

Statistics

For all statistical analyses, confirm that the following items are present in the figure legend, table legend, main text, or Methods section.

n/a	Confirmed	
☐	☒	The exact sample size (*n*) for each experimental group/condition, given as a discrete number and unit of measurement
☐	☒	A statement on whether measurements were taken from distinct samples or whether the same sample was measured repeatedly
☐	☒	The statistical test(s) used AND whether they are one- or two-sided<br>*Only common tests should be described solely by name; describe more complex techniques in the Methods section.*
☒	☐	A description of all covariates tested
☐	☒	A description of any assumptions or corrections, such as tests of normality and adjustment for multiple comparisons
☐	☒	A full description of the statistical parameters including central tendency (e.g. means) or other basic estimates (e.g. regression coefficient) AND variation (e.g. standard deviation) or associated estimates of uncertainty (e.g. confidence intervals)
☐	☒	For null hypothesis testing, the test statistic (e.g. *F*, *t*, *r*) with confidence intervals, effect sizes, degrees of freedom and *P* value noted<br>*Give P values as exact values whenever suitable.*
☐	☒	For Bayesian analysis, information on the choice of priors and Markov chain Monte Carlo settings
☒	☐	For hierarchical and complex designs, identification of the appropriate level for tests and full reporting of outcomes
☐	☒	Estimates of effect sizes (e.g. Cohen's *d*, Pearson's *r*), indicating how they were calculated

Our web collection on statistics for biologists contains articles on many of the points above.

Software and code

Policy information about availability of computer code

Data collection	no softwares were used to collect the data
Data analysis	RNAseq: FastQC (https://www.bioinformatics.babraham.ac.uk/projects/fastqc) ; STAR (v2.5.3a); HTseq v0.7.2; DESeq2 v1.19.37; TopGO v2.44.0<br>Bisulfite sequencing analysis: Bismark-bowtie 2 (https://www.bioinformatics.babraham.ac.uk/projects/bismark/).<br>DMR calling: DMRcaller R package v1.22.0, bedtools v2.27.1, genomation R package v1.22.0

For manuscripts utilizing custom algorithms or software that are central to the research but not yet described in published literature, software must be made available to editors and reviewers. We strongly encourage code deposition in a community repository (e.g. GitHub). See the Nature Portfolio guidelines for submitting code & software for further information.

Data

Policy information about availability of data

All manuscripts must include a data availability statement. This statement should provide the following information, where applicable:

- Accession codes, unique identifiers, or web links for publicly available datasets
- A description of any restrictions on data availability
- For clinical datasets or third party data, please ensure that the statement adheres to our policy

The raw data have been deposited at Gene Expression Omnibus GEO (https://www.ncbi.nlm.nih.gov/geo/query/acc.cgi?acc=GSE186857). Bisulfite sequencing raw

data and bigwig files showing methylation rates (#methylated C/#total number of C) in the context of CHH, CHG and CpG, where H: is A, C or T in the WT, M23 and M25 are under the accession number GSE186855. The raw RNA sequencing data and the TPM counting table are under accession GSE186856.
All the code that has been used to generate the results in this paper is available from the lead contact upon request.

All the cell lines used in the study, including DNMT5:KOs, are available upon request.

Human research participants

Policy information about studies involving human research participants and Sex and Gender in Research.

Reporting on sex and gender	not applicable
Population characteristics	not applicable
Recruitment	not applicable
Ethics oversight	not applicable

Note that full information on the approval of the study protocol must also be provided in the manuscript.

Field-specific reporting

Please select the one below that is the best fit for your research. If you are not sure, read the appropriate sections before making your selection.

☒ Life sciences ☐ Behavioural & social sciences ☐ Ecological, evolutionary & environmental sciences

For a reference copy of the document with all sections, see nature.com/documents/nr-reporting-summary-flat.pdf

Life sciences study design

All studies must disclose on these points even when the disclosure is negative.

Sample size	Computational analysis used in this study handle sample size differences. For example, read counts in RNAseq were normalized by library sizes.
Data exclusions	no data were excluded from the analysis
Replication	For bisulfite sequencing one biological replicate has been used for the WT, two biological replicates for DNMT5:KOs (M23 and M25). Two technical replicates were pooled for each biological replicate.<br>For RNAseq, we sequenced two technical replicates for each sample.
Randomization	no computational analysis recquiering randomization were used in this study.
Blinding	no tests recquiering blinding were used in this study.

Reporting for specific materials, systems and methods

We require information from authors about some types of materials, experimental systems and methods used in many studies. Here, indicate whether each material, system or method listed is relevant to your study. If you are not sure if a list item applies to your research, read the appropriate section before selecting a response.

Materials & experimental systems

n/a	Involved in the study	
☒	☐	Antibodies
☒	☐	Eukaryotic cell lines
☒	☐	Palaeontology and archaeology
☒	☐	Animals and other organisms
☒	☐	Clinical data
☒	☐	Dual use research of concern

Methods

n/a	Involved in the study	
☒	☐	ChIP-seq
☒	☐	Flow cytometry
☒	☐	MRI-based neuroimaging
